# Effect of a High Proportion of Rye in Compound Feed for Reduction of *Salmonella* Typhimurium in Experimentally Infected Young Pigs

**DOI:** 10.3390/microorganisms8111629

**Published:** 2020-10-22

**Authors:** Bussarakam Chuppava, Volker Wilke, Clara Berenike Hartung, Amr Abd El-Wahab, Richard Grone, Andreas von Felde, Josef Kamphues, Christian Visscher

**Affiliations:** 1 Institute for Animal Nutrition, University of Veterinary Medicine Hannover, Foundation, Bischofsholer Damm 15, D-30173 Hanover, Germany; bussarakam.chuppava@tiho-hannover.de (B.C.); volker.wilke@tiho-hannover.de (V.W.); clara.berenike.hartung@tiho-hannover.de (C.B.H.); josef.kamphues@tiho-hannover.de (J.K.); 2 Department of Nutrition and Nutritional Deficiency Diseases, Faculty of Veterinary Medicine, Mansoura University, Mansoura 35516, Egypt; amrwahab5@mans.edu.eg; 3 KWS LOCHOW GmbH, Ferdinand von Lochowstrasse 5, D-29303 Bergen, Germany; richard.grone@kws.com (R.G.); andreas.vonfelde@kws.com (A.v.F.)

**Keywords:** rye, wheat, *Salmonella*, foodborne pathogen, zoonosis, public health, swine, pig

## Abstract

Public health concerns and the potential for food-borne zoonotic transmission have made *Salmonella* a subject of surveillance programs in food-producing animals. Forty-two piglets (25 d of age and initially 7.48 kg) were used in a 28 d infection period to evaluate the effects of a high proportion of rye on reducing *Salmonella* Typhimurium. Piglets were divided into two diet groups: control diet (wheat 69%) and experimental diet (rye 69%). After a one-week adaptation period, all piglets were orally infected with *Salmonella* Typhimurium (10^7^ log CFU/mL; 2mL/pig). *Salmonella* in fecal shedding were evaluated at day 1, 3, 5, 7 and then weekly after infection. At the end of the experimental period (at day 28 after infection), the piglets were euthanized to sample feces, cecal digesta contents and ileocecal lymph nodes to determine the bacterial counts of *Salmonella*. The results suggest that the bacterial counts in the experimental group fed rye diets showed evidence of reducing *Salmonella* fecal shedding from day 14 onwards and decreasing the number of *Salmonella* in cecal digesta. However, the translocation of *Salmonella* in ileocecal lymph nodes was not affected. Furthermore, feed intake, weight gain and feed conversion did not differ between the groups (*p* > 0.05).

## 1. Introduction

Salmonellosis is primarily a zoonosis and interventions are possible at any stage from farm to fork [1]. *Salmonella enterica* serovar Typhimurium is strongly associated with pigs, with infections in humans caused by contaminated pork and meat products, causing an impact on public health [2,3]. In Germany, 33% of acquired infections or 13,529 cases of salmonellosis were reported in 2018 [4], making salmonellosis the second most commonly reported bacterial gastrointestinal disease in Germany after *Campylobacter* enteritis [4].

With regard to reducing the number of *Salmonella* cases in the feed-to-food chain, it has frequently been demonstrated that the use of antimicrobial agents in food animals favors the development of resistance among foodborne pathogens like *Salmonella* spp. [5]. Since antimicrobial resistance is on the rise, therefore, there is an urgent need for new strategies to focus on the reduction of *Salmonella* in the intestinal tract by competitive binding of bacteria and support of intestinal function to reduce colonization [6]. Due to their bacteriostatic and bacteriolytic properties, short-chain fatty acids such as formic or butyric acid and their derivatives were reported in previous studies, these being widely used to control *Salmonella* in the pork production chain already at the farm level [7,8,9].

Feeding concepts for fattening pigs are often based on wheat, triticale and maize [10]. However, due to the improvements attained through rye, the concentrations of anti-nutritional factors such as alkylresorcinols and trypsin inhibitors, as well as ergot poisoning, have been reduced [11,12]. In recent years, intensive studies on rye focused on the use of high proportions of rye in rations with high dietary fiber contents, which had various positive effects [13,14,15,16]. The effects of rye in pig feeding, which are of particular interest, are induced by the fraction of non-starch polysaccharides (NSPs) [17]. Rye is by far the most fiber-rich grain [16], with high levels of arabinoxylans and fructans, which can only be broken down by microorganisms of the large intestine, opening up opportunities for a targeted influence on the fermentation pattern in the direction of forced butyrate formation in the cecum [16,18]. Consequently, butyrate is the main metabolic product of anaerobic bacteria fermentation in the intestine, which can contribute to intestinal health [19]. It is tempting to speculate that modifying the gut microbiota to produce more butyrate could reduce invasive *Salmonella* infections in animals [7,8,9,19]. Butyrate can interact with *Salmonella* proteins and, as a result, the invasion capacity decreases. Furthermore, it can reduce the penetration of *Salmonella* into intestinal epithelial cells and thus reduce the exposure of pigs to *Salmonella* [9,19,20,21].

In the present study, the focus of interest was the question of whether the effects of a new concept, especially high rye content in the feed mixture of young pigs, could reduce the prevalence of *Salmonella* in fecal shedding. In addition, cecal contents of pigs were examined because the invasion of *Salmonella* takes place primarily in this location [22]. Furthermore, possible translocation of the pathogen from the intestine into the animal body by detecting of *Salmonella* in the lymph nodes was investigated. This is of crucial importance due to the risk of *Salmonella* entering the food chain. The issue of animal health on growing performance is also of great concern.

## 2. Materials and Methods

### 2.1. Ethical Statement

The experiments took place in the biosafety level 2 infection stable (S2) of the Institute for Animal Nutrition, University of Veterinary Medicine Hannover, Foundation, Germany in accordance with German regulations and were approved by the Ethics Committee of Lower Saxony for the Care and Use of Laboratory Animals, LAVES (Niedersächsisches Landesamt für Verbraucherschutz und Lebensmittelsicherheit; reference: 33.8-42502-04-19/3257; announcement dated 27.11.2019).

### 2.2. Animal and Housing

A total of forty-two weaned pigs came from piglet production from the Farm for Education and Research in Ruthe, University of Veterinary Medicine Hannover, Foundation (N = 42). The piglets were divided into three consecutive trials (T) with fourteen animals each (n = 14 per trial), at the age of 24.9 ± 0.7 days. The animals were selected for the formation of two comparable groups in each trial (control: n = 7 and experimental: n = 7) on the basis of the following criteria: weight (control: 7.48 ± 1.11 kg and experimental: 7.48 ± 1.20 kg), sex ratio (female/male castrated) and sow (siblings distributed to both groups). At the time of grouping, the animals were weighed and marked with an individual ear tag.

The stable was divided into fourteen compartments, the piglets were housed individually. The pen dimensions measured 1.1 m × 2.2 m per animal. Each animal pen was equipped with a nipple drinker, a tiltable feeding trough, a 1 m^2^ rubber mat as a lying surface and an infrared lamp hanging above the animal. Room temperature was recorded with a data logger (EBI 20-T1, Xylem Analytics Germany Sales GmbH & Co. KG, Weilheim, Germany) during the respective trial period (24.5 ± 0.8 °C) and the lighting program in the stable was set at a twelve-hour day and night rhythm, so that the light was switched on from 07:00 to 19:00.

Before beginning the trials, the stable and all materials were disinfected and tests were carried out to confirm that they were free of *Salmonella* species contamination. To rule out *Salmonella* contamination of the feed and thus entry thereof via the feed into the infection stable, the feed batches were examined in advance for *Salmonella*. To prevent cross-contamination between the individual pens, each pen was equipped with its own utensils (brooms, trays, spatulas). Protective clothing, disposable boot covers and gloves could be changed before entering the corresponding area of the stable. The positions of the groups in the stable were changed between the trials.

### 2.3. Diets

The composition of diets is described in Table 1. The piglets were allocated to two groups (control: N = 21, n = 7 and experimental: N = 21, n = 7) and fed ad libitum with the complete pelleted feed depending on whether wheat served as the control group (contained 69% wheat) or rye (experimental group; contained 69% rye, Table 1), according to the previous result from Wilke [14].

Feed chemical composition as well as particle size distribution of the diets are summarized in Table 2. Diets were analyzed by standard procedures in accordance with the official methods of the Verband Landwirtschaftlicher Untersuchungs- und Forschungsanstalten (VDLUFA [23]). The standardized methods of the Department of Animal Science, Aarhus University, Denmark were used to evaluate the NSP and arabinoxylans levels. Feed particle size distribution was assessed by a wet-sieve method of Wolf et al. [24].

### 2.4. Experimental Design

**Figure 1:**
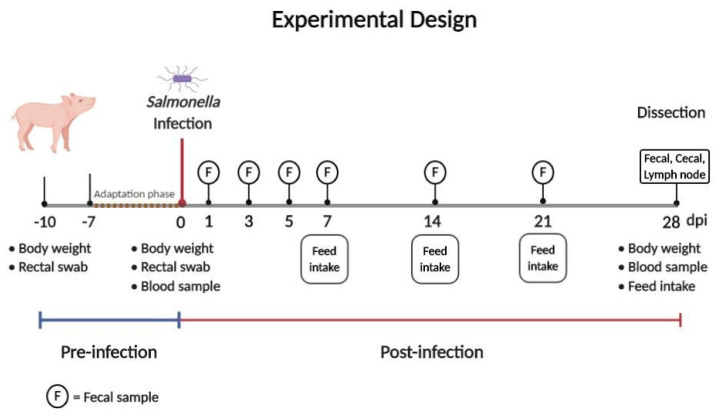
Overview concerning the time and sample plan for *Salmonella* diagnostics.

The *Salmonella* Typhimurium strain in this study was obtained from the field study [25]: *S.* Typhimurium (antigenic formula: 1,4,5,12:i:1,2, Phage type DT 193). Three consecutive trials of the infection experiment followed in accordance with the same investigation scheme (Figure 1). For the experiment carried out after a one-week adaptation period, all piglets were orally infected (day 0) with 2 mL of a broth containing ~ 1 × 10^7^ colony-forming units (CFU) *S.* Typhimurium/animal directly into the throat using a drencher as already described [26].

After the infection, animals were used in a 28-day experiment to determine the effects of control (69% wheat) or experimental group (69% rye) in complete pelleted feed, and to determine a possible effect of the diets on the duration of and level of *Salmonella* excretion. Fecal samples of the piglets were examined microbiologically for *Salmonella* species at defined time points (at 1, 3, 5, 7, 14, 21 and 28 days post-infection (dpi), as shown in Figure 1) to evaluate the status of the *Salmonella* infection. At the end of the experimental period (28 dpi), cecal chyme and lymph nodes were tested for *Salmonella*.

During the experimental phase, performance parameters, i.e., feed intake, were recorded on a weekly basis (Figure 1). To prevent cross-contamination between the animals during the infection, animals were weighed before the infection (0 dpi) and again at the end of the experiment (28 dpi; Figure 1). Corresponding parameters, average body weight gain (BWG) and feed conversion ratio (FCR) were determined.

In accordance with standardized *Salmonella* diagnostics, a blood sample was taken from each animal on the day of the experimental infection and at the end of the experiment (Figure 1). Serum was examined serologically for specific antibodies against *Salmonella*; *Salmonella* antibody ELISA (IDEXX Swine *Salmonella* antibody Test, IDEXX Europe B.V., Hoofddorp, the Netherlands) was used. The cut-off value was ≥10% optical density (OD).

### 2.5. Bacteriological Analyses and Salmonella Detection

Samples were taken for bacteriological analysis, and bacterial counts were determined for *Salmonella* detection. All test sections were carried out following the DIN EN ISO 6579-1 and 6579-2 guidelines [27,28]. Briefly, all collected samples were first placed for pre-enrichment in buffered peptone water (BPW; Oxoid Deutschland GmbH, Wesel, Germany), the ratio of which was 1:10, based on the volume of fecal or cecal chyme sample or the mass of the lymph node. For the next process of qualitative analysis, an incubation overnight at 37 °C took place. Each BPW-inoculated sample was placed with three drops (100 µL/drop) on modified semisolid Rappaport–Vassiliadis agar (MSRV; Oxoid Deutschland GmbH, Wesel, Germany) and incubated for a further 24 h (h) at 41 °C. After the incubation period, the evaluation was carried out macroscopically. If the result was positive, a white-grayish cloudy swarming zone spread over the entire agar resulting from the drops. Suspected *Salmonella* samples were also spread on the second selective nutrient media, xylose lysine deoxycholate agar (XLD; Oxoid Deutschland GmbH, Wesel, Germany) and Brilliance Salmonella agar (Oxoid Deutschland GmbH, Wesel, Germany) so that a confirmed result could be read after a 24 h incubation period.

In addition, *Salmonella* quantitative analysis of the fecal and cecal contents was performed as previously described [28,29]. Due to the high number of samples, a most probable number (MPN) method was used. One gram of the homogenized fecal or cecal content and 9 mL of BPW were vortexed. The bacterial count of the sample material was determined by a serial dilution with BPW in a deep well block (Sarstedt AG & Co, Nümbrecht, Germany). Quantitative proof of the dilution steps in a microtiter plate (Sarstedt AG & Co, Nümbrecht, Germany) was carried out in triplicate, as already described in detail [26]. After a 24 h incubation at 37 °C, the total volume of each well was transferred to another deep well block filled with MSRV agar and incubated for 24 h at 41 °C. The results were confirmed by cultural cultivation on Brilliance Salmonella agar, and subsequently the number of bacteria was calculated by an MPN software program [30]. To confirm the experimentally used *Salmonella* strain, salmonella-like colonies on Brilliance Salmonella agar were finally excluded by serotyping.

### 2.6. Statistical Analysis

The SAS software package version 7.1 (SAS Institute, Cary, NC, USA) was used for the statistical evaluation. The evaluation was carried out in cooperation with the Institute for Biometry, Epidemiology and Information Processing of the University of Veterinary Medicine Hannover, Foundation. Measurements such as mean values and standard deviations were calculated for the descriptive statistics. *Salmonella* results and performance parameters, i.e., body weight and feed intake, were analyzed at the level of the individual animal.

For evaluating the quantitative parameters between the control and experimental groups, two-way analysis of variance (ANOVA) with group and trial as independent factors was conducted. For analysis differences concerning distribution in qualitative parameters (*Salmonella* detection in cecal content, etc.), the chi-squared homogeneity test was used. Differences with a significant level of *p* < 0.05 were considered significant.

## 3. Results

### 3.1. Salmonella Prevalence

**Figure 2:**
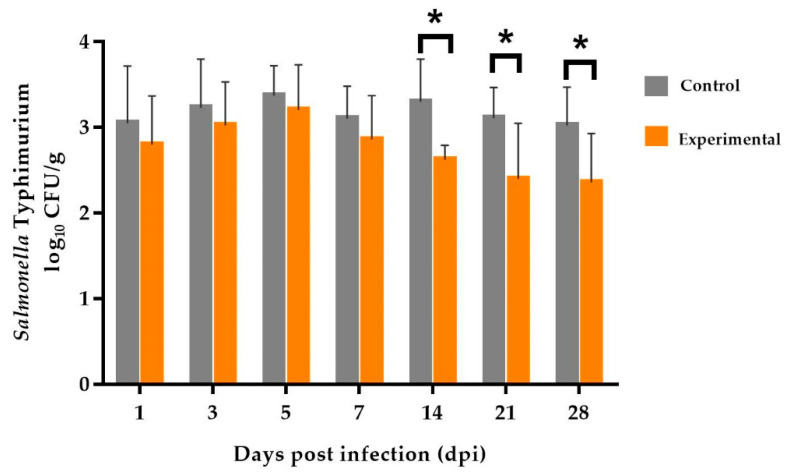
Counts of *Salmonella* (log_10_ CFU/g fecal sample) of piglets after an experimental oral infection with *S.* Typhimurium in different diets. Means of bacterial counts between the control (69% wheat) and experimental (69% rye) groups differ significantly (* *p* < 0.05).

The results of this study demonstrated that at the beginning of the post-infection period (1, 3, 5, 7 dpi), all the pigs were colonized. There were no significant differences in mean bacterial counts (log_10_ ± SD) of *Salmonella* in the feces between the wheat and rye groups. A peak in *Salmonella* shedding occurred at day 5 after infection in our study. However, at day 14 onwards after infection, feeding a pellet diet containing 69% rye was associated with a significantly lower bacterial count of *Salmonella* in fecal samples than in those taken from the group fed with 69% wheat in the diet, as shown in Figure 2 and in detail in Table S1 (control and experimental, 14 dpi: 3.30 ^a^ ± 0.50 and 2.62 ^b^ ± 0.18 (*p* < 0.001), 21 dpi: 3.11 ^a^ ± 0.36 and 2.40 ^b^ ± 0.65 (*p* < 0.001), 28 dpi: 3.02 ^a^ ± 0.45 and 2.36 ^b^ ± 0.57 (*p* = 0.001), respectively).

After oral infection with *S.* Typhimurium, different diets had significant influence on the counts of *Salmonella* in the cecal content (Table 3). The bacterial counts in the cecal content also differed significantly (log_10_ CFU/g; control group: 3.34 ^a^ ± 0.50, experimental: 3.08 ^b^ ± 0.56; *p* = 0.038; Table 3).

However, cecal content and ileocecal lymph node samples were qualitatively *Salmonella* positive (control: 100% and 61.9% and experimental: 100% and 66.7%, respectively; Table 3), but no differences could be seen between the two groups.

Three consecutive experiments were conducted using piglets from different sows. The results of the counts of *Salmonella* (log_10_ CFU/g fecal sample) for different trials and different diets are shown in Table 4. After initial statistical evaluations, a marked effect for the factor trial was shown, with significant effects at 1 dpi (*p* < 0.001), 7 dpi (*p* = 0.036), 21 dpi (*p* = 0.001) and 28 dpi (*p* < 0.001). An effect of diet seems to be present for days 14 (*p* < 0.001), 21 (*p* < 0.001) and 28 (*p* < 0.001) after infection. In addition, a bacterial count for *Salmonella* in cecal content was significantly affected by the diet (*p* = 0.035). Interestingly, the two-way analysis of variance (ANOVA) showed significant interactions between the factors diet and trial, which were observed for days 5 and 7 (*p* = 0.011 and *p* = 0.041, respectively; Table 4). On the other hand, there was not a significant effect of the type of diet by the trial interaction at day 3 after infection (*p* = 0.188; Table 4).

### 3.2. Serological Test for Specific Antibodies Against Salmonella

Optical density (OD)% was used to determine the *Salmonella* antibody status in blood samples from piglets. In both groups, on the day before infection, all of the pigs were seronegative. At day 28 after infection, regarding the number of positive blood samples from piglets, there was no significant difference in seroprevalence detected between the groups (*p* > 0.05; Table S2).

### 3.3. Animal Performance

**Figure 3:**
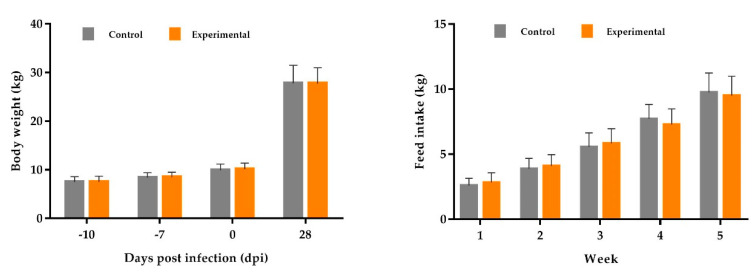
Performance data during the entire experimental period.

As a result of the evaluations carried out, there were no significant differences in the performance parameters between the groups. During the experimental period, before and after infection, there were no significant differences in the body weight (BW), as shown in Figure 3 and in detail in Table 5 and Supplementary Table S3. Furthermore, feed intake was measured individually for the entire trial period. Differences between the groups regarding the mean weekly feed intake (FI) were not statistically significant (control and experimental; mean FI at week 5 (21–28 dpi, in kg): 9.78 ^a^ ± 1.52 and 9.40 ^a^ ± 1.48; *p* = 0.136; Figure 3 and Table S3).

Table 5 summarizes the performance data during the experiments with experimental *Salmonella* infection from the day before infection (0 dpi) to the day of dissection (28 dpi). No significant differences were noted between the groups for BW at each time point (0 dpi (*p =* 0.085) and 28 dpi (*p =* 0.980)) and body weight gain (BWG; Table 5). The average daily weight gain (ADG) in the control group was about 639 g and in the experimental group, 629 g (Table 5). Similarly, the feed conversion ratio (FCR = feed requirement in kg per kg BWG) was not significantly affected between the wheat and rye diet groups (1.50 and 1.51, respectively).

## 4. Discussion

In recent years, the interest in rye has increased in terms of its good sustainability [31]. However, the use of rye in large amounts in diets for pig production is not typical because it can be associated with ergot poisoning [32]. On the other hand, rye has a high dietary fiber content [17], the fraction of NSPs in rye being more fermentable for monogastric animals than is the case for other cereals [13]. Nowadays, varieties of rye have been developed which reduce its susceptibility to ergot contamination; therefore, we can include rye in diets for swine [11,33].

The present study shows results of a feeding concept in young pigs fed with a high proportion of rye, and its effects on *Salmonella* prevalence, along with analyzing its effects on performance. In terms of *Salmonella* shedding, a significant reduction in the number of bacterial counts of *Salmonella* was seen in feces from 14 day onwards when considering the entire infection period for the experimental group. Feeding a diet containing 69% rye to young pigs may play a significant role in reducing *Salmonella* shedding after infection. In addition, the prevalence of *Salmonella* was found to be significantly lower in the cecal chyme of pigs fed with rye diets. Hence, if there was a reduction in the incidence of *Salmonella* in feces of pigs, which is the most likely source of contamination in the pig production chain, then it may help to reduce the risk of human salmonellosis.

To our knowledge, fermented rye has been previously studied to control *Salmonella* in nursery pigs [34]. Fabà et al. [34] reported that pigs fed with organic acids combined with fermented rye had reduced *S.* Typhimurium shedding compared to pigs fed with organic acids plus coated butyrate (3.11 and 3.87 log_10_ CFU/g, respectively) over the 21 d period post-challenge with 10^9^ CFU/mL. However, the pigs in our study were infected with 10^7^ CFU of *S.* Typhimurium, so comparison across infection studies is difficult. In this respect, an infection dosage in the present study could provide more opportunities to determine the effects of diet, which corroborates with other studies [8,35,36]. The *S.* Typhimurium oral challenge resulted in an effective infection in our study. At the beginning of the trial, all the pigs in the study showed a *Salmonella*-negative result. Then, after oral infection, nearly all infected animals excreted *Salmonella* until the end of the study. In addition, it is assumed that the challenge dose in practice or natural infection is often established with low or moderate numbers of *Salmonella* when compared with our study [36]. However, the scientific literature on the usefulness of a high proportion of rye in diets in a dry and pelleted form for *Salmonella* reduction in livestock animals, specifically young pigs, is scarce.

A reduction in *Salmonella* shedding in pigs in this study was possibly related to the components in rye such as dietary fiber, including NSPs. Compared to the total concentration of NSPs and arabinoxylans between groups, the mean concentration was higher in rye in the present study. The concentration of total NSPs was 123 g/kg dry matter (DM) in wheat and 140 g/kg DM in rye. In addition, the mean concentration of arabinoxylans was 63 g/kg DM and 74 g/kg DM in wheat and rye diets, respectively (Table 2). The concentrations of carbohydrate fractions in wheat and rye were in general agreement with those values of previous studies [17,18]. The NSP content of rye has traditionally been put forward to explain the effects on gut health benefits for pigs, especially arabinoxylans (8–9%) and fructans (approximately 3% and range up to about 6%) [33,37]. Rye has greater concentrations of arabinoxylans and fructans than other cereal grains [38,39] that can be changed by microorganisms to butyrate in the digestive tract [10,17].

According to Wilke [14], the effects in terms of nutritional physiology have been evaluated. In the group with 69% rye in the diet, significantly higher concentrations of lactate in the anterior digestive tract were found, as well as an increased entry of fermentable organic substances in the large intestine (approx. 35% higher butyrate concentrations) in the ingesta of cecum and colon, the preferred colonization area of *Salmonella,* in comparison to those animals given a wheat-based diet [22]. How rye affects *Salmonella* colonization is unclear. Consequently, the antimicrobial effects of lactate and butyrate seem to play an important role against *Salmonella* [14]. High butyrate concentrations in the hindgut have been found when a high amount of rye in diets is given to young pigs [14]. The focus on the mechanism of action of butyrate in reducing *Salmonella* has been explained in various studies [7,9,19,40,41]; similar mechanisms possibly play a role in our study. Further investigations are needed to better elucidate the *Salmonella* shedding reduction and other health-promoting mechanisms potentially associated with rye on the physiological processes in the gastrointestinal tract and the stability of the intestinal microbiota of pigs.

Less fecal *Salmonella* excretion indicates less colonization of *Salmonella* in the intestine and reduced severity of the infection [42,43]. In addition, in our study, the lymph node was used to confirm colonization. A translocation of *Salmonella* from the intestine into the adjacent ileocecal lymph nodes was investigated in all experimental runs. The piglets were totally *Salmonella* positive at the time of dissection (in cecal chyme), but positive evidence in lymph nodes was found in twenty-seven of a total of forty-two animals (control: n = 13; experimental: n = 14). Less is known about the role of rye in the translocation effect of *Salmonella* in the pig model. However, in previous experiments with poultry, this was determined with other pathogens such as *Escherichia coli* and *Clostridium perfringens* [44,45]. When observing the elevated bacteria translocation while feeding a diet rich in NSPs [44], the prolonged transit time of the intestinal contents possibly induces proliferation of pathogenic bacteria. Moreover, Tellez et al. [45] also reported that a rye diet negatively influences bacterial translocation and intestinal viscosity in poultry. Nevertheless, the viscosity does not pose a large problem in swine as much as in poultry [46].

The outcome of the current study showed that the use of compound feeds with higher dietary proportions of rye in young pigs caused no significant differences in performance, i.e., average body weight gain, feed intake and feed conversion ratio compared with those of animals fed with generally high amounts of wheat. Similar results were obtained by Grone [15] and Wilke [14], who observed no negative impact on growth performance when young pigs were fed diets containing a high proportion of rye. Previous studies have shown positive effects of feeding rye on performance data [46,47,48]. Among the beneficial effects of the dietary fiber components in rye, it can provide energy to the pig mostly via the digestion of starch and fermentation of fiber in the hindgut. Moreover, the NSPs in rye may promote greater butyrate production and improve intestinal health [33]. Furthermore, scientific studies have evaluated the feed digestibility in pigs concerning diets containing rye [48]. The rye-based diets had higher digestibility coefficients for dry matter, crude protein and gross energy than barley when rye was included in a young growing pig diet at 60% [49]. Thacker et al. [46] suggest that rye may have much more potential in pig performance when administered in pellet form. Furthermore, to improve feed efficiency and average daily gain, the NSP enzyme would be recommended for diets containing high rye levels [47]. Contrary to the present results, a previous study on the use of rye in pig feeding reported that if more than 50% of the barley or wheat in a swine diet was replaced with rye, a significant reduction in pig performance was observed [50]. The reason for rye being less palatable is that it is more bitter than other small grains such as wheat [51]. Alkylresorcinols in rye may be responsible for the unpleasant taste, but reduced levels of alkylresorcinols are found in hybrid rye [11].

## 5. Conclusions

The results of this study support the view that a high proportion of rye might contribute to reducing *Salmonella* shedding via feces in young pigs. In addition, overall, the animal performance was good, so that no relevant differences could be observed between the rye and wheat diets. It can be suggested that up to 70% rye in compound pelleted feed poses no problem for animal health and welfare, additionally decreasing possible contamination with *Salmonella.* Another advantage that puts rye in a better light today than in the past is its sustainable benefits, namely, it needs less fertilizer, demands substantially less water, can reduce CO_2_ emissions in pork production in comparison with wheat [52] and its price, as it is an inexpensive raw feed material compared to other cereals [11].

Nonetheless, to our knowledge, this is the first time that high amounts of rye were used in a *Salmonella* challenge in a young pig model. Furthermore, our results can provide useful information, prompting further studies of a field trial on a commercial pig farm or large-scale farming study over a longer period, not only in young pigs, but also in all phases of pig production. However, we cannot consider all factors when only a small group of pigs are observed. Therefore, research is needed to better understand the gut and fecal microbiota composition underlying the effects observed when a high proportion of rye is fed to pigs.

## Figures and Tables

**Table 1:** Composition of diets during the experiment as percentages.

Feedstuffs	Control	Experimental
Wheat	69.0	
Rye	-	69.0
Soybean meal *	11.5	11.5
Barley	10.0	10.0
Potato protein	5.10	4.90
Calcium carbonate	1.00	0.90
Monocalcium phosphate	0.90	1.00
Fat (soybean oil) *	0.50	0.50
Sodium chloride	0.35	0.40
Feed additives **	1.65	1.80

* Soybean meal (steam heated, with soapstock) made from genetically modified soybeans. ** Additives (per kg as fed); nutritional additives: vitamin A (12,000 IU), vitamin D3 (2000 IU), vitamin E (150 mg), copper from copper-(II)-glycinate chelate hydrate (4 mg), copper from copper-(II)-sulfate pentahydrate (110 mg), manganese from manganese glycine manganese chelate hydrate (35 mg), manganese from manganese-(II)-oxide (45 mg), zinc from glycine zinc chelate hydrate (40 mg), zinc from zinc oxide (80 mg), iron from iron-(II)-sulfate monohydrate (200 mg), iodine from calcium iodate anhydrous (2.0 mg), and selenium from sodium selenite (0.40 mg); zootechnical additives: 5.0 × 10^9^ CFU *Saccharomyces cerevisiae*.

**Table 2:** Feed chemical composition of control and experimental diets (g/kg dry matter).

Item	Control	Experimental
Dry matter (g/kg as fed)	897	903
Crude ash	51.4	54.2
Crude protein	205	213
Ether extract	28.0	23.6
Crude fiber	28.7	28.2
Starch	516	461
Sugar	41.0	61.0
Nitrogen-free extract (NfE) ^1^	584	681
Metabolizable energy (ME) ^2^(MJ/kg DM)	15.6	15.5
Calcium	9.21	9.26
Phosphorus	5.74	6.42
Arginine	10.4	10.8
Cysteine	3.73	4.01
Isoleucine	7.98	8.01
Leucine	14.5	14.3
Lysine	14.4	15.1
Methionine	4.64	5.71
Phenylalanine	9.89	9.72
Threonine	7.97	9.60
Valine	9.62	10.0
Non-starch polysaccharides (NSPs) *	123	140
Arabinoxylans *	63.0	74.0
Particle size (in%)		
>2 mm	3.55	2.83
>1–≤2 mm	28.1	25.3
≥0.2–≤1 mm	30.5	30.4
<0.2 mm	37.9	41.5

^1^ Nitrogen-free extract (NfE) = dry matter—(crude ash + ether extract + crude fiber + crude protein). ^2^ Metabolizable energy (ME) calculated from the specified raw nutrient content. * NSPs and arabinoxylans were analyzed by the Department of Animal Science, Aarhus University, Denmark.

**Table 3:** Number of *Salmonella*-positive samples from cecal content and ileocecal lymph nodes at dissection after oral infection with *S.* Typhimurium.

Sample	*Salmonella* Test	Control	Experimental
Cecal content	Quantitative log_10_ CFU/g	3.34 ^a^ ± 0.50	3.08 ^b^ ± 0.56
	Qualitative (n_pos_/n_total_)	21 ^a^/21	21 ^a^/21
Lymph nodes	Qualitative (n_pos_/n_total_)	13 ^a^/21	14 ^a^/21

^a,b^ Means in a row with different superscripts differ significantly between groups (*p* < 0.05). n_pos_ = number of positive samples, n_total_ = number of total samples.

**Table 4:** Counts of *Salmonella* (log_10_ CFU/g fecal sample) of piglets after an experimental oral infection with *Salmonella* Typhimurium for different trials and different diets.

Time Point	Trial						*p*-Value		
	Trial 1		Trial 2		Trial 3				
	Diet								
	Con	Exp	Con	Exp	Con	Exp	Trial	Diet	Trial x Diet
1 dpi	2.46 ^b^ ± 0.40	2.46 ^b^ ± 0.50	2.97 ^b^ ± 0.53	2.46 ^b^ ± 0.10	3.71 ^a^ ± 0.36	3.49 ^a^ ± 0.11	**<0.001**	0.089	0.206
3 dpi	3.00 ^a^ ± 0.58	3.14 ^a^ ± 0.73	3.20 ^a^ ± 0.77	2.63 ^a^ ± 0.18	3.49 ^a^ ± 0.11	3.29 ^a^ ± 0.20	0.054	0.187	0.188
5 dpi	3.29 ^ab^ ± 0.23	3.66 ^a^ ± 0.55	3.51 ^ab^ ± 0.43	2.94 ^b^ ± 0.36	3.31 ^ab^ ± 0.38	3.00 ^b^ ± 0.40	0.111	0.176	**0.011**
7 dpi	3.01 ^ab^ ± 0.39	3.23 ^a^ ± 0.64	2.94 ^b^ ± 0.41	2.54 ^b^ ± 0.10	3.34 ^a^ ± 0.25	2.80 ^b^ ± 0.42	**0.036**	0.059	**0.041**
14 dpi	3.40 ^a^ ± 0.85	2.71 ^b^ ± 0.28	3.23 ^a^ ± 0.21	2.57 ^b^ ± 0.08	3.29 ^a^ ± 0.20	2.57 ^b^ ± 0.08	0.524	**<0.001**	0.981
21 dpi	2.71 ^ab^ ± 0.28	2.00 ^c^ ± 1.05	3.40 ^a^ ± 0.00	2.57 ^b^ ± 0.08	3.23 ^ab^ ± 0.21	2.63 ^b^ ± 0.18	**0.001**	**<0.001**	0.807
28 dpi (Dissection)	2.43 ^b^ ± 0.08	1.97 ^c^ ± 0.90	3.34 ^a^ ± 0.15	2.54 ^b^ ± 0.10	3.29 ^a^ ± 0.20	2.57 ^b^ ± 0.08	**<0.001**	**<0.001**	0.478
Cecalcontent	3.48 ^a^ ± 0.86	3.09 ^b^ ± 0.97	3.43 ^a^ ± 0.08	2.99 ^b^ ± 0.23	3.43 ^a^ ± 0.08	3.17 ^b^ ± 0.21	0.883	**0.035**	0.888

^a,b,c^ Different subscripts within a row mark significant differences (*p* < 0.05). Con (control): animals fed with 69% wheat compound pelleted diet; Exp (experimental): animals fed with 69% rye compound pelleted diet.

**Table 5:** Performance data during the experiments with experimental *Salmonella* infection, in kg.

Parameter	Group		*p*-Value
	Control (*n* = 21)	Experimental (*n* = 21)	
Body weight (0 dpi)	9.89 ^a^ ± 1.28	10.1 ^a^ ± 1.31	0.085
Body weight (28 dpi)	27.8 ^a^ ± 3.72	27.7 ^a^ ± 3.28	0.980
BWG/piglet (0–28 dpi)	17.9 ^a^ ± 2.54	17.6 ^a^ ± 2.29	0.847
BWG/group (0–28 dpi)	376 ^a^ ± 2.54	370 ^a^ ± 2.29	0.229
ADG (g/d)	639 ^a^ ± 3.12	629 ^a^ ± 2.75	0.079
Total feed intake (0–28 dpi)	564 ^a^ ± 3.93	560 ^a^ ± 4.11	0.126
FCR (kg/kg)	1.50 ^a^ ± 0.07	1.51 ^a^ ± 0.13	0.786

^a^ Means ± SD within the same row with same superscripts do not differ significantly (*p* > 0.05). Control: animals fed with 69% wheat compound pelleted diet; Experimental: animals fed with 69% rye compound pelleted diet. dpi = day after infection; BWG = body weight gain, ADG = average daily weight gain, FCR = feed conversion ratio.

## Supplementary Materials

The following are available online at https://www.mdpi.com/2076-2607/8/11/1629/s1, Table S1: Counts of *Salmonella* (log_10_ CFU/g fecal sample) of piglets after an experimental oral infection with *Salmonella* Typhimurium for different diets. Table S2: S*almonella* antibody status in serum of piglets in control and experimental groups. Table S3: Performance data during the entire experimental period.
