# Supplementary material for: Effect of a High Proportion of Rye in Compound Feed for Reduction of Salmonella Typhimurium in Experimentally Infected Young Pigs
Source: Microorganisms. 2020 Oct 22;8(11):1629. doi: 10.3390/microorganisms8111629 (PMC7690436; doi:10.3390/microorganisms8111629)
Supplement: Supplementary file 1 [file microorganisms-08-01629-s001.pdf]

Supplementary materials

Supplementary Table S1. Counts of *Salmonella* (log_{10} CFU/g fecal sample) of piglets after an experimental oral infection with *Salmonella* Typhimurium for different diets.

Group	*Salmonella* test		Collection time						
			1 dpi	3 dpi	5 dpi	7 dpi	14 dpi	21 dpi	28 dpi
Control	Qualitative (n_{pos}/n_{total})		21/21	21/21	21/21	21/21	21/21	21/21	21/21
	Quantitative log_{10} CFU/g	mean	3.05[a] ± 0.67	3.23[a] ± 0.57	3.37[a] ± 0.35	3.10[a] ± 0.38	3.30[a] ± 0.50	3.11[a] ± 0.36	3.02[a] ± 0.45
		min	2.0	2.0	2.6	2.4	2.4	2.4	2.4
		max	4.4	4.4	4.4	3.6	5.0	3.4	3.4
Experimental	Qualitative		21/21	21/21	21/21	21/21	21/21	20/21	20/21
	Quantitative log_{10} CFU/g	mean	2.80[a] ± 0.57	3.02[a] ± 0.51	3.20[a] ± 0.54	2.86[a] ± 0.51	2.62[b] ± 0.17	2.40[b] ± 0.65	2.36[b] ± 0.57
		min	2.0	2.4	2.6	2.4	2.4	0.0	0.0
		max	3.6	4.4	4.6	4.4	3.2	3.4	2.6
	p -Value		0.206	0.217	0.229	0.089	< 0.001	< 0.001	0.001

[a,b] Means in a column with different superscripts differ significantly between group ($p < 0.05$). Control: animals fed with 69% wheat compound pelleted diet; Experimental: animals fed with 69% rye compound pelleted diet. dpi = day post infection.

Supplementary Table S2. *Salmonella* antibody status in serum of piglets in control and experimental groups (n_{pos}/n_{total}).

Collection time	Group		*p*-value
	Control (n = 21)	Experimental (n = 21)	
Before infection (0 dpi)	0[a]/21	0[a]/21	0.834
End of the experiment (28 dpi)	15[a]/21	13[a]/21	0.466

Seropositive: optical density (OD) ≥10%. n_{pos} = number of positive samples, n_{total} = number of total samples. Control: animals fed with 69% wheat compound pelleted diet; Experimental: animals fed with 69% rye compound pelleted diet.

Supplementary Table S3. Performance data during the entire experimental period, in kg.

Parameter		Group	
		Control (n = 21)	Experimental (n = 21)
Body weight	10 days before infection	7.48[a] ± 1.11	7.48[a] ± 1.20
	7 days before infection	8.39[a] ± 1.01	8.44[a] ± 1.03
Feed intake	week 1	2.63[a] ± 0.68	2.80[a] ± 0.68
	week 2	4.01[a] ± 0.82	3.97[a] ± 0.88
	week 3	5.68[a] ± 1.10	5.66[a] ± 1.17
	week 4	7.54[a] ± 1.24	7.40[a] ± 1.23
	week 5	9.78[a] ± 1.52	9.40[a] ± 1.48

[a] Means ± SD within the same row with same superscripts do not differ significantly ($p > 0.05$). Control: animals fed with 69% wheat compound pelleted diet; Experimental: animals fed with 69% rye compound pelleted diet.
